# 8-Methyl-2-oxo-4-(thio­phen-2-yl)-1,2,5,6,7,8-hexa­hydro­quinoline-3-carbonitrile

**DOI:** 10.1107/S160053681202836X

**Published:** 2012-06-30

**Authors:** Abdullah M. Asiri, Hassan M. Faidallah, Alaa Anwar Ahmad Saqer, Seik Weng Ng, Edward R. T. Tiekink

**Affiliations:** a Chemistry Department, Faculty of Science, King Abdulaziz University, PO Box 80203, Jeddah, Saudi Arabia; b Center of Excellence for Advanced Materials Research (CEAMR), King Abdulaziz University, PO Box 80203, Jeddah 21589, Saudi Arabia; c Department of Chemistry, University of Malaya, 50603 Kuala Lumpur, Malaysia

## Abstract

In the title compound, C_15_H_14_N_2_OS, the pyridinone ring in the fused-ring system is nearly planar (r.m.s. deviation = 0.011 Å) and the cyclo­hexene ring has a twisted half-boat conformation with the methyl­ene C atom adjacent to the methine C atom deviating by 0.592 (7) Å from the plane defined by the remaining five atoms (r.m.s. deviation = 0.108 Å). The thienyl ring is disordered over two almost coplanar positions of opposite orientation in a 0.649 (4):0.351 (4) ratio, and forms dihedral angles of 51.4 (3) (major component) and 54.2 (3)°, respectively, with the pyridinone ring. In the crystal, inversion-related mol­ecules associate *via* an eight-membered {⋯HNCO}_2_ synthon and these are linked into a linear supra­molecular chain along the *a* axis by weak π–π inter­actions that occur between centrosymmetrically related pyridinone rings [centroid–centroid distance = 3.889 (2) Å].

## Related literature

For background to the cardiotonic and anti-inflammatory properties of this class of compounds, see: Behit & Baraka (2005 ▶); Girgis *et al.* (2007 ▶). For a related structure, see: Asiri *et al.* (2011 ▶).
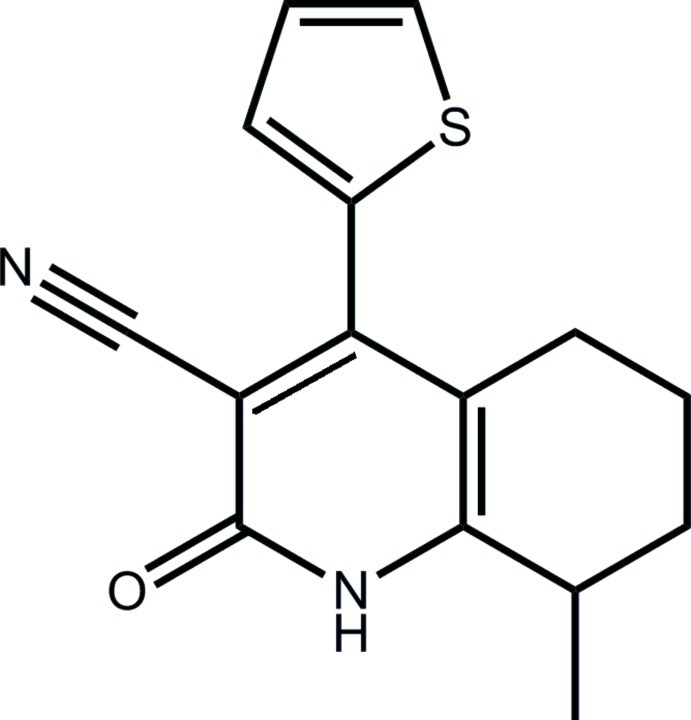


## Experimental

### 

#### Crystal data

C_15_H_14_N_2_OS
*M* *_r_* = 270.34
Triclinic, 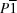

*a* = 7.6443 (3) Å
*b* = 9.6909 (5) Å
*c* = 9.9852 (5) Å
α = 67.003 (5)°
β = 80.869 (4)°
γ = 76.108 (4)°
*V* = 659.26 (5) Å^3^
*Z* = 2
Mo *K*α radiation
μ = 0.24 mm^−1^
*T* = 100 K
0.30 × 0.20 × 0.05 mm

#### Data collection

Agilent SuperNova Dual diffractometer with an Atlas detector
Absorption correction: multi-scan (*CrysAlis PRO*; Agilent, 2012 ▶) *T*_min_ = 0.798, *T*_max_ = 1.000
9707 measured reflections
3041 independent reflections
2356 reflections with *I* > 2σ(*I*)
*R*_int_ = 0.031

#### Refinement

*R*[*F* ^2^ > 2σ(*F* ^2^)] = 0.088
*wR*(*F* ^2^) = 0.230
*S* = 1.07
3041 reflections
185 parameters
33 restraints
H-atom parameters constrained
Δρ_max_ = 1.02 e Å^−3^
Δρ_min_ = −0.78 e Å^−3^

### 

Data collection: *CrysAlis PRO* (Agilent, 2012 ▶); cell refinement: *CrysAlis PRO*; data reduction: *CrysAlis PRO*; program(s) used to solve structure: *SHELXS97* (Sheldrick, 2008 ▶); program(s) used to refine structure: *SHELXL97* (Sheldrick, 2008 ▶); molecular graphics: *ORTEP-3* (Farrugia, 1997 ▶) and *DIAMOND* (Brandenburg, 2006 ▶); software used to prepare material for publication: *publCIF* (Westrip, 2010 ▶).

## Supplementary Material

Crystal structure: contains datablock(s) global, I. DOI: 10.1107/S160053681202836X/xu5572sup1.cif

Structure factors: contains datablock(s) I. DOI: 10.1107/S160053681202836X/xu5572Isup2.hkl

Supplementary material file. DOI: 10.1107/S160053681202836X/xu5572Isup3.cml

Additional supplementary materials:  crystallographic information; 3D view; checkCIF report

## Figures and Tables

**Table 1:** Hydrogen-bond geometry (Å, °)

*D*—H⋯*A*	*D*—H	H⋯*A*	*D*⋯*A*	*D*—H⋯*A*
N1—H1*n*⋯O1^i^	0.88	1.94	2.801 (4)	168

Symmetry code: (i) 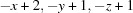.

**Fig. 1.**
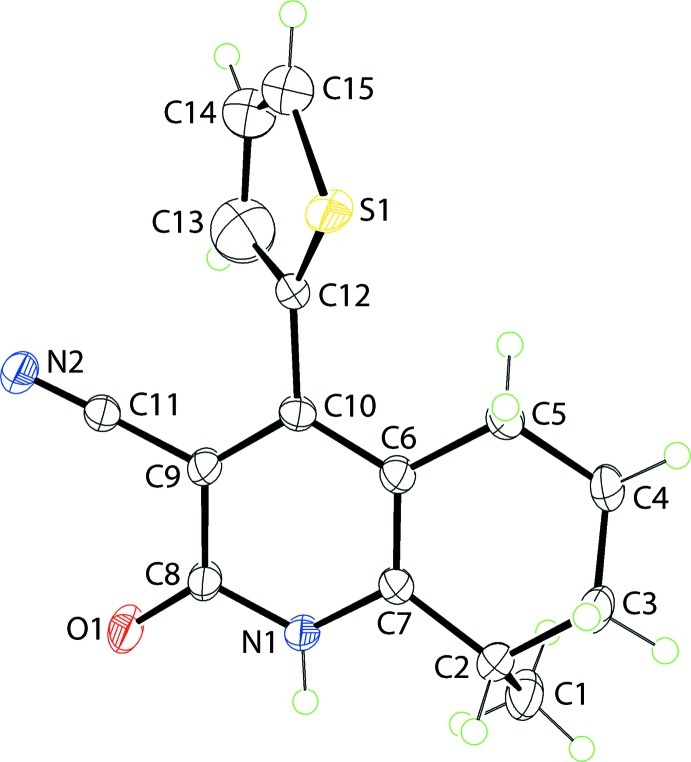
The molecular structure of (I) showing the atom-labelling scheme and displacement ellipsoids at the 50% probability level.

**Fig. 2.**
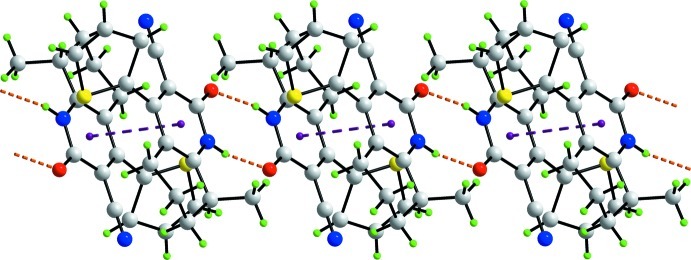
A view of the supramolecular chain along the a axis in (I) mediated by N—H···O and π—π interactions, shown as orange and purple dashed lines, respectively.

**Fig. 3.**
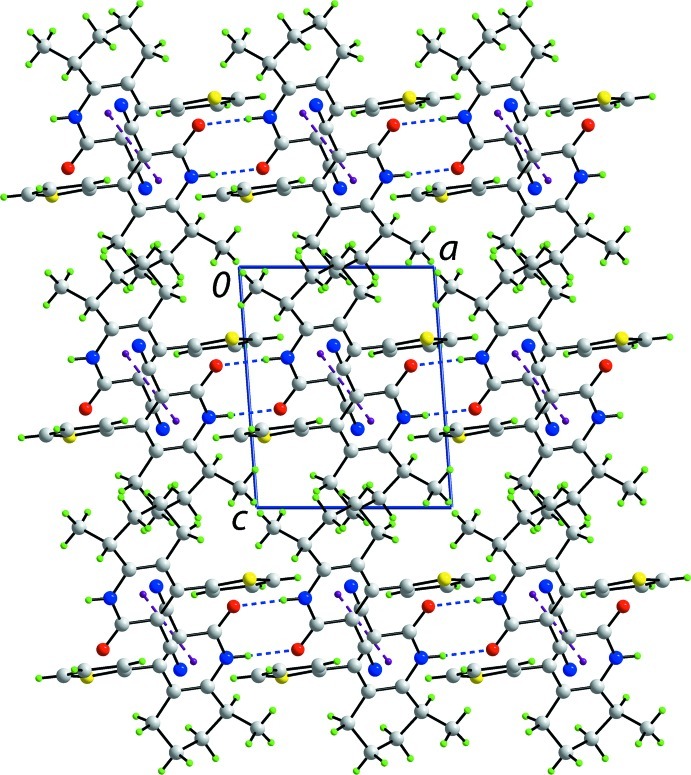
A view in projection down the b axis of the unit-cell contents of (I). The N—H···O and π—π interactions are shown as orange and purple dashed lines, respectively.

## supplementary crystallographic information

### Comment

The title compound (I) is a member of a series of cyano-pyridinones that have been evaluated for their cardiotonic (Behit & Baraka, 2005) and anti-inflammatory (Girgis *et al.*, (2007) properties. Herein, the crystal and molecular structures of (I) are described.

In (I), Fig. 1, the pyridyl ring in the fused ring system is planar [r.m.s. deviation = 0.011 Å] and the cyclohexene ring has a twisted half-boat conformation with the methylene-C3 atom lying -0.592 (7) Å above the plane defined by the remaining five atoms [r.m.s. deviation = 0.108 Å]. There are two orientations of the thienyl ring [co-planar, with a dihedral angle of 4.4 (4)°, and of opposite orientations] both of which are inclined with respect to the pyridyl ring, forming dihedral angles of 51.4 (3)° [major component] and 54.2 (3)°, respectively. The molecular structure of (I) resembles that found in a literature structure with the exception of the C2—C3 conformation which is fused to a benzene ring (Asiri *et al.*, 2011).

The familiar eight-membered centrosymmetric amide {···HNCO}_2_ synthon is observed in the crystal packing, Table 1. These are connected into a linear supramolecular chain along the *a* axis by π—π interactions that occur between centrosymmetrically related pyridyl rings [inter-centroid distance = 3.889 (2) Å for symmetry operation 1 - *x*, 1 - *y*, 1 - *z*], Fig. 2. Chains assemble into layers in the *ab* plane and stack along the *c* axis being separated by hydrophobic interactions, Fig. 3.

### Experimental

A mixture of the thiophene-2-carboxaldehyde (1.1 g, 0.01 *M*), 2-methylcyclohexanone (1.12 g, 0.01 *M*), ethyl cyanoacetate (1.1 g, 0.01 *M*) and ammonium acetate (6.2 g, 0.08 *M*) in absolute ethanol (50 ml) was refluxed for 6 h. The reaction mixture was allowed to cool. The formed precipitate was filtered, washed with water, dried and recrystallized from ethanol as yellow crystals, *M*. pt: 525–527 K. Yield: 72%.

### Refinement

Carbon-bound H-atoms were placed in calculated positions [C—H = 0.95–0.99 Å, *U*_iso_(H) = 1.2–1.5*U*_eq_(C)] and were included in the refinement in the riding model approximation. The N-bound H-atom was treated similarly with N—H = 0.88 Å and with *U*_iso_(H) = 1.2*U*_eq_(N). The thienyl ring is disordered over two positions [co-planar and opposite orientation] in a 0.649 (4):0.351 (4) ratio. Pairs of 1,2-related distances were restrained to within 0.01 Å of each other, and the rings were restrained to be within 0.01 Å of a plane. The anisotropic displacement parameters, restrained to be nearly isotropic, of the primed atoms were set to those of the unprimed ones. The maximum and minimum residual electron density peaks of 1.02 and -0.78 e Å^-3^, respectively, were located 0.14 Å and 0.39 Å from the C15' and C13 atoms, respectively.

### Crystal data

**Table:**

C_15_H_14_N_2_OS	*Z* = 2
*M**_r_* = 270.34	*F*(000) = 284
Triclinic, *P*1	*D*_x_ = 1.362 Mg m^−^^3^
Hall symbol: -P 1	Mo *K*α radiation, λ = 0.71073 Å
*a* = 7.6443 (3) Å	Cell parameters from 3729 reflections
*b* = 9.6909 (5) Å	θ = 2.3–27.5°
*c* = 9.9852 (5) Å	µ = 0.24 mm^−^^1^
α = 67.003 (5)°	*T* = 100 K
β = 80.869 (4)°	Plate, yellow
γ = 76.108 (4)°	0.30 × 0.20 × 0.05 mm
*V* = 659.26 (5) Å^3^	

### Data collection

**Table:**

Agilent SuperNova Dual diffractometer with an Atlas detector	3041 independent reflections
Radiation source: SuperNova (Mo) X-ray Source	2356 reflections with *I* > 2σ(*I*)
Mirror monochromator	*R*_int_ = 0.031
Detector resolution: 10.4041 pixels mm^-1^	θ_max_ = 27.6°, θ_min_ = 2.3°
ω scan	*h* = −9→9
Absorption correction: multi-scan (*CrysAlis PRO*; Agilent, 2012)	*k* = −12→12
*T*_min_ = 0.798, *T*_max_ = 1.000	*l* = −12→12
9707 measured reflections	

### Refinement

**Table:**

Refinement on *F*^2^	Primary atom site location: structure-invariant direct methods
Least-squares matrix: full	Secondary atom site location: difference Fourier map
*R*[*F*^2^ > 2σ(*F*^2^)] = 0.088	Hydrogen site location: inferred from neighbouring sites
*wR*(*F*^2^) = 0.230	H-atom parameters constrained
*S* = 1.07	*w* = 1/[σ^2^(*F*_o_^2^) + (0.0899*P*)^2^ + 1.5187*P*] where *P* = (*F*_o_^2^ + 2*F*_c_^2^)/3
3041 reflections	(Δ/σ)_max_ = 0.001
185 parameters	Δρ_max_ = 1.02 e Å^−^^3^
33 restraints	Δρ_min_ = −0.78 e Å^−^^3^

### Special details

**Table:**

Geometry. All e.s.d.'s (except the e.s.d. in the dihedral angle between two l.s. planes) are estimated using the full covariance matrix. The cell e.s.d.'s are taken into account individually in the estimation of e.s.d.'s in distances, angles and torsion angles; correlations between e.s.d.'s in cell parameters are only used when they are defined by crystal symmetry. An approximate (isotropic) treatment of cell e.s.d.'s is used for estimating e.s.d.'s involving l.s. planes.
Refinement. Refinement of *F*^2^ against ALL reflections. The weighted *R*-factor *wR* and goodness of fit *S* are based on *F*^2^, conventional *R*-factors *R* are based on *F*, with *F* set to zero for negative *F*^2^. The threshold expression of *F*^2^ > σ(*F*^2^) is used only for calculating *R*-factors(gt) *etc*. and is not relevant to the choice of reflections for refinement. *R*-factors based on *F*^2^ are statistically about twice as large as those based on *F*, and *R*- factors based on ALL data will be even larger.

### Fractional atomic coordinates and isotropic or equivalent isotropic displacement parameters (Å^2^)

**Table:**

	*x*	*y*	*z*	*U*_iso_*/*U*_eq_	Occ. (<1)
O1	0.8432 (4)	0.6552 (5)	0.4105 (4)	0.0607 (13)	
N1	0.7871 (4)	0.4615 (4)	0.6240 (4)	0.0359 (9)	
H1n	0.8984	0.4118	0.6160	0.043*	
N2	0.4540 (5)	0.9325 (4)	0.3270 (4)	0.0359 (9)	
C1	0.9207 (6)	0.2563 (6)	0.9181 (5)	0.0405 (11)	
H1A	1.0051	0.3102	0.8423	0.061*	
H1B	0.9847	0.1546	0.9765	0.061*	
H1C	0.8702	0.3144	0.9813	0.061*	
C2	0.7691 (5)	0.2402 (5)	0.8473 (4)	0.0303 (9)	
H2	0.8246	0.1755	0.7884	0.036*	
C3	0.6307 (6)	0.1585 (5)	0.9600 (5)	0.0337 (10)	
H3A	0.5668	0.1120	0.9147	0.040*	
H3B	0.6941	0.0752	1.0422	0.040*	
C4	0.4947 (6)	0.2674 (5)	1.0173 (5)	0.0361 (10)	
H4A	0.5584	0.3154	1.0611	0.043*	
H4B	0.4121	0.2101	1.0945	0.043*	
C5	0.3854 (5)	0.3914 (5)	0.8953 (4)	0.0300 (9)	
H5A	0.3015	0.3458	0.8667	0.036*	
H5B	0.3120	0.4703	0.9318	0.036*	
C6	0.5042 (5)	0.4659 (4)	0.7629 (4)	0.0219 (7)	
C7	0.6793 (5)	0.3935 (4)	0.7432 (4)	0.0244 (8)	
C8	0.7363 (5)	0.6006 (5)	0.5159 (5)	0.0367 (11)	
C9	0.5537 (5)	0.6763 (4)	0.5364 (4)	0.0252 (8)	
C10	0.4413 (5)	0.6133 (4)	0.6569 (4)	0.0204 (7)	
C11	0.4972 (5)	0.8193 (5)	0.4214 (4)	0.0258 (8)	
C12	0.2592 (5)	0.7030 (4)	0.6738 (4)	0.0311 (9)	
S1	0.0646 (2)	0.6528 (2)	0.7033 (2)	0.0426 (7)	0.649 (4)
S1'	0.2014 (7)	0.8647 (5)	0.6683 (4)	0.043*	0.351 (4)
C13	0.260 (2)	0.8772 (14)	0.6570 (7)	0.069 (4)	0.649 (4)
H13	0.3559	0.9306	0.6399	0.082*	0.649 (4)
C13'	0.079 (2)	0.627 (2)	0.6905 (12)	0.069*	0.351 (4)
H13'	0.0714	0.5285	0.6971	0.082*	0.351 (4)
C14	0.0544 (12)	0.9255 (9)	0.6783 (8)	0.0461 (18)	0.649 (4)
H14	0.0031	1.0261	0.6752	0.055*	0.649 (4)
C14'	−0.072 (2)	0.7647 (18)	0.6921 (14)	0.046*	0.351 (4)
H14'	−0.1961	0.7600	0.6992	0.055*	0.351 (4)
C15	−0.0505 (11)	0.8293 (9)	0.7006 (7)	0.0432 (18)	0.649 (4)
H15	−0.1783	0.8545	0.7141	0.052*	0.649 (4)
C15'	−0.0217 (15)	0.8881 (17)	0.6833 (11)	0.043*	0.351 (4)
H15'	−0.1023	0.9788	0.6852	0.052*	0.351 (4)

### Atomic displacement parameters (Å^2^)

**Table:**

	*U*^11^	*U*^22^	*U*^33^	*U*^12^	*U*^13^	*U*^23^
O1	0.0203 (15)	0.071 (3)	0.0354 (18)	0.0116 (15)	0.0094 (13)	0.0234 (17)
N1	0.0157 (15)	0.041 (2)	0.0255 (18)	0.0083 (14)	0.0040 (13)	0.0050 (15)
N2	0.0270 (18)	0.035 (2)	0.0275 (18)	0.0013 (15)	−0.0005 (14)	0.0029 (15)
C1	0.026 (2)	0.045 (3)	0.031 (2)	−0.0043 (18)	−0.0045 (17)	0.0055 (19)
C2	0.026 (2)	0.027 (2)	0.025 (2)	0.0028 (15)	−0.0006 (15)	−0.0016 (16)
C3	0.031 (2)	0.0230 (19)	0.032 (2)	−0.0038 (16)	−0.0018 (17)	0.0049 (16)
C4	0.030 (2)	0.034 (2)	0.025 (2)	−0.0049 (17)	0.0056 (16)	0.0047 (17)
C5	0.0189 (18)	0.030 (2)	0.029 (2)	−0.0054 (15)	0.0050 (15)	0.0000 (16)
C6	0.0180 (17)	0.0239 (18)	0.0205 (17)	−0.0050 (13)	−0.0003 (13)	−0.0046 (14)
C7	0.0197 (17)	0.0267 (19)	0.0202 (17)	−0.0022 (14)	0.0012 (13)	−0.0039 (15)
C8	0.0157 (18)	0.045 (2)	0.024 (2)	0.0044 (16)	0.0023 (14)	0.0071 (18)
C9	0.0174 (17)	0.0285 (19)	0.0209 (18)	−0.0013 (14)	−0.0021 (13)	−0.0013 (15)
C10	0.0151 (16)	0.0236 (17)	0.0213 (17)	−0.0047 (13)	−0.0007 (13)	−0.0067 (14)
C11	0.0164 (17)	0.031 (2)	0.0221 (18)	−0.0023 (14)	0.0006 (13)	−0.0034 (16)
C12	0.0238 (19)	0.035 (2)	0.0189 (18)	0.0049 (16)	0.0027 (14)	−0.0018 (16)
S1	0.0090 (7)	0.0483 (10)	0.0447 (10)	−0.0033 (6)	−0.0002 (5)	0.0084 (7)
C13	0.069 (4)	0.069 (4)	0.068 (4)	−0.0132 (15)	−0.0044 (13)	−0.0250 (18)
C14	0.046 (2)	0.045 (2)	0.043 (2)	−0.0018 (12)	0.0006 (12)	−0.0171 (13)
C15	0.040 (2)	0.042 (2)	0.041 (2)	−0.0030 (12)	0.0010 (12)	−0.0128 (13)

### Geometric parameters (Å, º)

**Table:**

O1—C8	1.246 (5)	C6—C10	1.429 (5)
N1—C7	1.366 (5)	C8—C9	1.436 (5)
N1—C8	1.371 (5)	C9—C10	1.381 (5)
N1—H1n	0.8800	C9—C11	1.433 (5)
N2—C11	1.148 (5)	C10—C12	1.477 (5)
C1—C2	1.516 (6)	C12—S1'	1.503 (5)
C1—H1A	0.9800	C12—S1	1.622 (4)
C1—H1B	0.9800	C12—C13	1.632 (13)
C1—H1C	0.9800	C12—C13'	1.668 (17)
C2—C7	1.513 (5)	S1—C15	1.717 (8)
C2—C3	1.530 (5)	S1'—C15'	1.656 (11)
C2—H2	1.0000	C13—C14	1.537 (15)
C3—C4	1.510 (6)	C13—H13	0.9500
C3—H3A	0.9900	C13'—C14'	1.541 (18)
C3—H3B	0.9900	C13'—H13'	0.9500
C4—C5	1.524 (6)	C14—C15	1.304 (12)
C4—H4A	0.9900	C14—H14	0.9500
C4—H4B	0.9900	C14'—C15'	1.309 (15)
C5—C6	1.511 (5)	C14'—H14'	0.9500
C5—H5A	0.9900	C15—H15	0.9500
C5—H5B	0.9900	C15'—H15'	0.9500
C6—C7	1.377 (5)		
			
C7—N1—C8	125.4 (3)	O1—C8—N1	121.6 (4)
C7—N1—H1n	117.3	O1—C8—C9	124.0 (4)
C8—N1—H1n	117.3	N1—C8—C9	114.4 (3)
C2—C1—H1A	109.5	C10—C9—C11	123.1 (3)
C2—C1—H1B	109.5	C10—C9—C8	122.1 (3)
H1A—C1—H1B	109.5	C11—C9—C8	114.9 (3)
C2—C1—H1C	109.5	C9—C10—C6	119.8 (3)
H1A—C1—H1C	109.5	C9—C10—C12	118.5 (3)
H1B—C1—H1C	109.5	C6—C10—C12	121.7 (3)
C7—C2—C1	111.3 (4)	N2—C11—C9	178.5 (4)
C7—C2—C3	111.3 (3)	C10—C12—S1'	130.3 (4)
C1—C2—C3	112.2 (4)	C10—C12—S1	129.1 (3)
C7—C2—H2	107.3	S1'—C12—S1	100.5 (3)
C1—C2—H2	107.3	C10—C12—C13	113.3 (6)
C3—C2—H2	107.3	S1—C12—C13	117.5 (6)
C4—C3—C2	111.4 (3)	C10—C12—C13'	119.2 (8)
C4—C3—H3A	109.4	S1'—C12—C13'	110.3 (8)
C2—C3—H3A	109.4	C13—C12—C13'	127.1 (11)
C4—C3—H3B	109.4	C12—S1—C15	92.6 (4)
C2—C3—H3B	109.4	C12—S1'—C15'	102.2 (6)
H3A—C3—H3B	108.0	C14—C13—C12	95.8 (9)
C3—C4—C5	110.8 (4)	C14—C13—H13	132.1
C3—C4—H4A	109.5	C12—C13—H13	132.1
C5—C4—H4A	109.5	C14'—C13'—C12	99.6 (13)
C3—C4—H4B	109.5	C14'—C13'—H13'	130.2
C5—C4—H4B	109.5	C12—C13'—H13'	130.2
H4A—C4—H4B	108.1	C15—C14—C13	120.5 (8)
C6—C5—C4	112.3 (3)	C15—C14—H14	119.8
C6—C5—H5A	109.2	C13—C14—H14	119.8
C4—C5—H5A	109.2	C15'—C14'—C13'	117.0 (15)
C6—C5—H5B	109.2	C15'—C14'—H14'	121.5
C4—C5—H5B	109.2	C13'—C14'—H14'	121.5
H5A—C5—H5B	107.9	C14—C15—S1	113.6 (6)
C7—C6—C10	118.1 (3)	C14—C15—H15	123.2
C7—C6—C5	120.2 (3)	S1—C15—H15	123.2
C10—C6—C5	121.7 (3)	C14'—C15'—S1'	110.9 (12)
N1—C7—C6	120.2 (3)	C14'—C15'—H15'	124.6
N1—C7—C2	114.9 (3)	S1'—C15'—H15'	124.6
C6—C7—C2	124.9 (3)		
			
C7—C2—C3—C4	−42.7 (5)	C9—C10—C12—S1'	−51.3 (6)
C1—C2—C3—C4	82.7 (5)	C6—C10—C12—S1'	127.3 (4)
C2—C3—C4—C5	62.8 (5)	C9—C10—C12—S1	128.4 (4)
C3—C4—C5—C6	−49.6 (5)	C6—C10—C12—S1	−52.9 (5)
C4—C5—C6—C7	19.3 (6)	C9—C10—C12—C13	−50.5 (4)
C4—C5—C6—C10	−159.4 (4)	C6—C10—C12—C13	128.2 (4)
C8—N1—C7—C6	−0.6 (7)	C9—C10—C12—C13'	123.6 (5)
C8—N1—C7—C2	−179.4 (4)	C6—C10—C12—C13'	−57.7 (5)
C10—C6—C7—N1	−0.8 (6)	C10—C12—S1—C15	−178.7 (4)
C5—C6—C7—N1	−179.5 (4)	S1'—C12—S1—C15	1.1 (3)
C10—C6—C7—C2	177.8 (4)	C13—C12—S1—C15	0.15 (17)
C5—C6—C7—C2	−1.0 (6)	C13'—C12—S1—C15	−155 (3)
C1—C2—C7—N1	65.3 (5)	C10—C12—S1'—C15'	175.7 (5)
C3—C2—C7—N1	−168.7 (4)	S1—C12—S1'—C15'	−4.1 (4)
C1—C2—C7—C6	−113.3 (5)	C13—C12—S1'—C15'	173.1 (11)
C3—C2—C7—C6	12.6 (6)	C13'—C12—S1'—C15'	0.42 (19)
C7—N1—C8—O1	179.8 (5)	C10—C12—C13—C14	178.9 (4)
C7—N1—C8—C9	0.7 (7)	S1'—C12—C13—C14	−3.2 (10)
O1—C8—C9—C10	−178.3 (5)	S1—C12—C13—C14	−0.1 (2)
N1—C8—C9—C10	0.8 (7)	C13'—C12—C13—C14	5.4 (5)
O1—C8—C9—C11	3.1 (7)	C10—C12—C13'—C14'	−175.7 (4)
N1—C8—C9—C11	−177.9 (4)	S1'—C12—C13'—C14'	0.2 (2)
C11—C9—C10—C6	176.3 (4)	S1—C12—C13'—C14'	25 (3)
C8—C9—C10—C6	−2.2 (6)	C13—C12—C13'—C14'	−2.5 (5)
C11—C9—C10—C12	−5.0 (6)	C12—C13—C14—C15	0.0 (4)
C8—C9—C10—C12	176.4 (4)	C12—C13'—C14'—C15'	−1.0 (5)
C7—C6—C10—C9	2.2 (5)	C13—C14—C15—S1	0.1 (6)
C5—C6—C10—C9	−179.1 (4)	C12—S1—C15—C14	−0.2 (4)
C7—C6—C10—C12	−176.4 (4)	C13'—C14'—C15'—S1'	1.3 (6)
C5—C6—C10—C12	2.3 (6)	C12—S1'—C15'—C14'	−1.0 (4)

### Hydrogen-bond geometry (Å, º)

**Table:**

*D*—H···*A*	*D*—H	H···*A*	*D*···*A*	*D*—H···*A*
N1—H1*n*···O1^i^	0.88	1.94	2.801 (4)	168

Symmetry code: (i) −*x*+2, −*y*+1, −*z*+1.
